# The experiences of preparation and engagement of educators in teaching e-portfolio

**DOI:** 10.1186/s12909-023-04642-1

**Published:** 2023-09-18

**Authors:** Fatemeh Keshmiri, Amir Houshang Mehrparvar

**Affiliations:** 1 Medical Education Department, Education Development Center, Shahid Sadoughi University of Medical Sciences, Yazd, Iran; 2 Occupational Medicine Department, Industrial Diseases Research Center, Shahid Sadoughi University of Medical Sciences, Yazd, Iran; 3 Occupational Medicine Department, Shahid Rahnemoon Hospital, Farrokhi Ave, Yazd, Iran

**Keywords:** Teaching, Portfolio, Faculty evaluation, Faculty Development, Scholarship of teaching and learning, Excellent teaching

## Abstract

**Introduction:**

A teaching e-portfolio is used to organize the collation and presentation of documents about teaching for the development and evaluation of educators. The current study was aimed at describing teaching e-portfolio components at Shahid Sadoughi University of Medical Sciences. As well, we examined the performance and experience of educators in engaging in the teaching e-portfolio.

**Materials and methods:**

This study was conducted at Shahid Sadoughi University in 2018–2022 in three main stages: (1) Development of the teaching e-portfolio; (2) Implementing teaching e-portfolio, and evaluating teaching documentation quantitatively; and (3) Exploration of educators’ experiences by a conventional content analysis introduced by Graneheim and Lundman. The teaching e-portfolio was developed from the perspective of the components, domains, and criteria of scholarship of teaching and learning. The teaching e-portfolio documented the educational activities of educators in 12 areas, including philosophy of education (1 activity), curriculum planning (4 activities), evaluation (7 activities), teaching and learning (1 activity), e-learning (1 activity), professional development in education (1 activity), scholarly activities (2 activities), mentoring and counseling (3 activities), educational leadership (2 activities), education research (6 activities), education reform project (1 activity), and production of scholarship of teaching and learning (13 activities). The educators recorded the documentation of educational activities in their teaching e-portfolio. Their documentation was reviewed by two peers. The reviewers delivered constructive feedback to improve the educators’ performance. The quantitative performance of educators in different activities in teaching e-portfolio was examined by descriptive tests (frequency and percentage). The experiences of educators were explored by the conventional content analysis approach which was introduced by Graneheim and Lundman.

**Results:**

In the present study, 148 educators registered in the teaching e-portfolio. A total of 1488 documents of educational activities were registered in the e-portfolio from 2018 to 2022, and 55.24% of the activities received feedback in the peer review process. The experience of participants was categorized into a theme “fear and hope in utilizing teaching e-portfolio”. This theme consisted of three categories: “motivational roadmap for personal and professional development in the future”, “concern about the consequences of continuous monitoring”, and “restriction of resources and capability as resistance sources”.

**Conclusion:**

The current study showed that the participation of educators in a teaching e-portfolio was at an acceptable level. Support systems and educational policies played an effective role in guiding educators to participate in educational development activities. The educators perceived the teaching e-portfolio as a two-faceted tool. Teaching e-portfolio can provide a road map for their personal and professional development to achieve excellent teaching. As well, the teaching e-portfolio was recognized as a tool for continuous performance monitoring and detection of the inefficiency of teaching quality activities. This perception, along with limited resources such as time, weak technological literacy, and difficulty in working with electronic devices and systems, led to resistance from educators to involve in teaching e-portfolio.

## Introduction

Teaching portfolios are applied as instruments for the development and evaluation of educators in universities [1–3]. The teaching portfolio is a compilation of documents related to educators’ activities of teaching quality in the classroom and the clinical environment. The purpose of a teaching portfolio is to document the full range of educators’ abilities during a specific period [4]. Many academic institutions use teaching portfolios to organize the collation and presentation of documents about teaching for the development and evaluation of faculty members [2, 5]. Reece et al. stated that a teaching portfolio documents an educator’s commitment to the scholarship of teaching-learning (SoTL), and its accomplishment [6]. The most critical functions of a teaching portfolio include showing the progress of educators to achieve excellent teaching, determining the level of knowledge about specialized subjects, and the type of innovative educational activities. As well, the ability and skill to solve educational problems, the method of designing and implementing the teaching-learning process, and the perception of educators towards education were recorded in a teaching portfolio [7–9].

Higher education systems have evolved portfolio step by step using concepts and new technologies in systems of faculty development and evaluation [10]. The teaching portfolios were developed under different conditions and for diverse purposes. Four types of portfolios were defined based on whether they are mandatory or voluntary and written for promotion or development. These four types include “dossier portfolio” (mandatory, written for promotion), “training portfolio” (mandatory, written for development), “reflective portfolio” (voluntary, written for promotion), and “personal development portfolio” (voluntary, written for development) [11].

The teaching portfolio stimulates educators’ professional development through self-assessment and reflection on their teaching practices [5, 12, 13]. Many universities use the portfolio to document educators’ activities and plan to develop their personal and professional abilities [14, 15]. The portfolios facilitate recognizing one’s professional strengths and weaknesses and reinforce their teaching skills. Preparing a teaching portfolio encourages educators to review teaching activities, reorganize priorities, reflect on strategies and methods, and plan for the future [9, 13].

Assessment of the quality of teaching is curial in academic institutes to make decisions about educators’ future in the university. Teaching e-portfolio was used as a reliable tool that provides valid evidence for formative and summative decisions [5]. Documentation of the quantity and quality of teaching is one of the key sources for evaluating educators’ performance [12, 13]. Evaluation of teaching effectiveness requires numerous sources of evidence. The teaching portfolio permits these multiple input sources to be comprised in performance evaluations of educators [5]. The teaching portfolio could be applied for both formative and summative assessments. The formative assessment uses evidence to improve the quality of educators’ teaching by reflection, and the summative assessment uses evidence to decide about promotion and tenure and shows the overall educator’s performance in the education field [5].

The issue of educational quality evaluation in universities of medical sciences is one of the concerns of managers of educational systems. The use of various fair and objective tools, and the use of targeted documents in a way that confirms the components of quality improvement in the educational system is considered a concern of the educators’ evaluation system [16]. In universities of medical sciences, due to the simultaneous duties of providing health services and teaching, the evaluation of faculty members faced challenges. Then, the design and application of appropriate tools in the evaluation of educators of medical universities so that assess all the expected roles of educators were required. With these tools, it was necessary to provide an integrated development and evaluation opportunity covering the main roles. In these universities, a typical curriculum vitae (CV) based on classical guidelines was used which mainly focused on research activities and providing health services. After a reform of the evaluation system in medical sciences faculties, a teaching portfolio was suggested to develop excellent teaching in the universities of medical sciences. Leading medical universities such as Harvard and John’s Hopkins created the clinician-educator track, where faculties were required to complete a teaching portfolio before applying for promotion [17].

The aim of using a teaching portfolio is to improve faculty performance and achievements in teaching [17, 18]. With the expansion of the need to use the teaching portfolio, one of the concerns of compiling the teaching portfolio in medical universities was the lack of consensus regarding its content and components. There are several diverse ideas in the literature [9, 17]. Lamki suggested a framework for a Medical Educator Teaching Portfolio (METP) that consisted of four parts: [1] evaluation, [2] personal professional development, [3] learning processes, and [4] an appendix [17]. Sidhu addressed the teaching portfolio as a reflection tool for anesthetists. He stated electronic portfolios are more portable and flexible compared to paper portfolios. The group with the highest benefit from a teaching portfolio is clinical educators because they can use it as a tool for contemplation of their teaching practice [19]. Further studies are required to develop teaching portfolio models that are compatible with the purpose and conditions of medical universities.

Although the use of portfolios in universities of North America and the UK has been reported in Sidhu’s study [19], based on our best knowledge, the experiences of these universities and the practical framework for evaluating the quality of education of educators in medical universities for teaching portfolios have been discussed in limited studies [17]. A teaching portfolio is considered an evaluation method that needs more studies to address its challenges in medical universities after its implementation. Recently, there has been a tendency to shift from paper portfolios to electronic portfolios and the focus of research has been changed to investigate the learning and experiences of educators who engaged in the teaching portfolio [10]. Quantitative and qualitative studies to assess the efficacy of e-portfolio from the viewpoint of different stakeholders are important. The exchange of experiences concerning education quality evaluation systems can help create a template with common and comprehensive features in medical sciences universities [17]. The results are helpful for managers of educational systems and managers of education quality development in universities of medical sciences.

At Shahid Sadoughi University of Medical Sciences, a teaching e-portfolio has been prepared and maintained since 2018. The current study was aimed at describing the experiences related to the preparation and engagement of educators in teaching e-portfolio. As well, we examined the performance and experiences of educators in engaging in the teaching e-portfolio. Accordingly, the research questions were:

 [1] How is the teaching e-portfolio described in the Shahid Sadoughi University of Medical Sciences?

 [2] What pros and cons do educators experience in using the teaching e-portfolio?

## Materials and methods

This study was conducted at Shahid Sadoughi University of Medical Sciences. The study used the perspective of the constructivist paradigm. A fundamental assumption of a constructivist paradigm is defined as an understanding of the world through the experiences of others [20]. The present study explored educators’ experiences that had prepared and maintained a teaching e-portfolio. In this study, we assumed that each educator had different experiences in engaging with a teaching portfolio, which influenced their perceptions.

### Study setting

This study was conducted at Shahid Sadoughi University of Medical Sciences in the Middle East region. In Iran, the system of medical education and providing health services has been integrated, and medical universities are in charge of both medical education and providing health services. Educators of this university are engaged in teaching basic medical sciences and clinical sciences. In this university, seven duties have been introduced for educators, and teaching, research, and providing health services are among the key duties. The evaluation of educators was mainly based on a survey by asking students’ opinions. The process of empowering the educators was performed uniformly according to the design of the training workshops by the managers of the Education Development Center (EDC) to improve the educators’ knowledge about teaching and evaluation methods. There were no supportive and structured opportunities for individualized personal and professional development in the role of teaching and educational scholarship to achieve excellent teaching.

The traditional educational strategies including teacher-centered, discipline-based, information-oriented basic medical science courses and hospital-based education, and opportunistic strategies in clinical education were used in the investigated university. The main teaching methods include lectures in classrooms and observation and gaining experience in teaching practical skills. Most of the educators were not familiar with the fields of scholarship of teaching and learning and did not have the experience of using its methods as a tool to develop their teaching and learning quality. Therefore, the need for a change in the educational system caused the teaching portfolio to be selected as a strategy to achieve the goal of change to gain excellent teaching in a way that creates an integrated system of evaluation and support for the development of educators’ competencies.

The use of teaching e-portfolio with formative and summative applications was implemented in this university for the first time in 2018. Before this, educators did not have the experience of using tools for recording their activities in the process of empowering and evaluating teaching. The design, implementation, and evaluation of the teaching e-portfolio were done in the EDC of the university.

### Conceptual framework

Teaching portfolio has been used for various purposes in educational systems and universities. Faculty development, formative and summative evaluation of the educators, and developing excellent teaching are considered as the most important uses of portfolios [21]. Pelger et al. explained a main conceptual framework for teaching portfolio which was achieving SoTL [21]. In the teaching portfolio, educators are supported to become excellent teachers. So, they will be encouraged to attain the expert level. Through this improvement, they can find the link between theory and practice and achieve SoTL. Pelger’s study illustrates the impacts of teaching portfolios on the improvement of academic teaching practice, professional learning, and the promotion of educators. By integrating a portfolio with peer feedback into the development and evaluation process of educators, a reflective approach can be encouraged within the community of academic teaching practice [21]. In line with Pelger’s study, Hamilton acknowledged that engaging in a teaching portfolio can scaffold the process of developing educational scholar roles among educators. The teaching portfolio provides opportunities for self-reflection and peer-feedback seeking from more experienced educators. It provides a situation for professional development planning connected to focused and evidenced feedback on practice [18]. In the development of a teaching e-portfolio in this study, the components, domains, and criteria of SoTL (six criteria introduced by Glassick) were considered [22].

This study used the electronic format of a portfolio. An e-portfolio is a tool consisting of a collection of activities of the educators including their attainments, experiences, and learning in a digital format which is supported by some complex processes including planning, discussing, getting feedback, and responding to it, etc. [10]. Quick and easy access to the portfolio, the ability to record documents in different formats such as audio and video files were advantages of e-portfolio. In addition, e-portfolio creates opportunities for informal reflection at different times, and planning for professional development without the time and place limitations by using the teaching portfolio [10]. It was one of the factors influencing the choice of e-portfolio in the present study.

This study was conducted in three main stages: (1) Development of the teaching e-portfolio; (2) Implementing teaching e-portfolio, and evaluating teaching documentation quantitatively; and (3) Exploration of educators’ experiences by a conventional content analysis introduced by Graneheim and Lundman. The stages are explained below:

### Stage 1: development of teaching e-portfolio

To develop a teaching e-portfolio, we reviewed ‘teaching responsibilities’ in the field of faculty evaluation and teacher roles by the following keywords: “faculty evaluation”, “educational documentation”, “faculty portfolio”, “teaching portfolio”, “excellent teaching”, “competency”, “teacher”, “scholarship of teaching and learning”, and “educational scholarship”, in PubMed, Science Direct, Scopus from 2000 till 2018 [2, 5, 6, 8, 9, 12, 13, 16, 23–35]. As well, we explored the viewpoints of different stakeholders related to teaching responsibilities (n = 25) through six focus group meetings. The results of the steps were discussed in an expert panel (where 12 experts in medical education (n = 2), educational leadership (n = 3), and executive management in the faculties (n = 7) participated). Sixty eight codes were explored in the steps. The findings were categorized into 12 areas: “the philosophy of education”, “curriculum planning”, “evaluation”, “teaching and learning”, “educational resources”, “e-learning”, “professional development in education”, “scholarly activities”, “mentoring and counseling”, “educational leadership”, “research in education”, “education reform project”, and “production of SoTL”. After that, the activities of each area were developed according to the expert opinion and SoTL components, domains, and criteria [22]. The expert opinion explored description statements for each activity, arrangement of the activities in order, completion of the supporting data, and formulation of the expected output according to different purposes. The teaching e-portfolio was developed in 7 steps that are shown in Table 1:

Step 1: To describe the teaching responsibilities.
Step 2: To select activities for each area of the teaching e-portfolio from the perspective of the SoTL concept.
Step 3: To prepare description statements for each activity.
Step 4: To arrange the activities in order.
Step 5: To compile the supporting data.
Step 6: To formulate the expected output according to different purposes.
Step 7: To produce a platform for a teaching e-portfolio.

**Table 1:** The process of development of teaching e-portfolio

**Step 1: To describe the teaching responsibilities**	This description should include a listing of the teaching area and guidelines for preparation and maintenance in the e-portfolio. The teaching responsibilities were categorized into: philosophy of education, curriculum planning, evaluation, teaching and learning, educational resources, e-learning, professional development in education, scholarly activities, mentoring and counseling, educational leadership, research in education, education reform project, and production of scholarship of teaching and learning.
**Step 2: To select activities for each area of the teaching e-portfolio in perspective of SoTL activities**	This step developed the content of the e-portfolio in different areas, including: Philosophy of education (1 activity), curriculum planning (4 activities), evaluation (7 activities), teaching and learning (1 activity), e-learning (1 activity), professional development in education (1 activity), scholarly activities (2 activities), mentoring and counseling (3 activities), educational leadership (2 activities), research in education (6 activities), education reform project (1 activity), and production of scholarship of teaching and learning (13 activities).
**Step 3: To prepare description statements for each activity**	In this step, the educational quality activities and its descriptions were developed. In addition, the standards of activities were defined—for instance, Glassick’s criteria for ‘the production of scholarship of teaching and learning activities”.
**Step 4: To arrange the activities in order**	In this step, the contents were arranged according to the formative and summative purposes of the teaching e-portfolio.
**Step 5: To compile the supporting data**	In this step, the eligible documentation for each activity and the format of reports were described. Supporting materials and valid content for these activities were formulated.
**Step 6: To formulate the expected output according to different purposes**	The teaching e-portfolio was designed to be used for different purposes, including self-assessment, and formative and summative evaluation. The framework of reports was structured. In this step, the components of formative assessment such as peer review, feedback, and reflection were prepared. As well, the mandates of summative assessment based on the promotion regulation in the university were considered in reporting systems of teaching e-portfolio.
**Step 7: To produce teaching e-portfolio**	The framework of the portfolio was formulated in an electronic system. The e-portfolio facilitates the accessibility to the portfolio, data entry, and reporting for different purposes.

### Stage 2: implementation of teaching e-portfolio and evaluation of the documentation quantitatively

For the implementation of the teaching portfolio, the introduction of the teaching portfolio, the application of the results, and how to work with it through various methods such as face-to-face meetings, online, and educational videos were conducted to assist educators in be familiar with the teaching portfolio. In the guidelines for evaluating the quality of the educational performance of educators, details related to areas, standards, related documents, and confirming authority were published. Moreover, instruction of peer review at the college and institute level was explained and informed to various stakeholders. The participation was voluntary, and to attract more educators’ participation, motivating factors were determined for educators to participate in the teaching portfolio. Instruction also explained the use of results’ formative and summative evaluation, such as tenure, promotion, and obtaining management positions in the field of education and the excellent teacher awards in educational festivals in the institute. The planning and implementation of this stage were done with the support of the managers of the educational system in the Vice-Chancellor of Education and EDC of the university.

To implement the e-portfolio in the university, training was held for stakeholders, including educators, and educational managers, through educational videos, training booklets, and face-to-face and virtual meetings. Also, the training of evaluators related to the evaluation process and criteria was done by an expert in health professions education.

The e-portfolio teaching platform is a web-based system and can be used on computers, tablets, smartphones, and Personal Digital Assistants (PDAs) with Windows, Android, and IOS operating systems. A dedicated profile was prepared for each educator on the teaching e-portfolio platform. Areas and activities were designed in this platform and educators could upload their documents in different formats of text, image, sound, video, etc. in the system.

The educators registered their documents of teaching activities in the teaching e-portfolio. The documents were peer-reviewed by two trained reviewers. To implement the formative approach in this process, peer feedback was and conducted in the e-portfolio. All documentation was reviewed by two peers in the process of formative assessment and delivered constructive feedback to improve the educator’s performance. The educators could select their activities to be considered in the summative assessment of the promotion process.

The recorded documentation of educators in different activities in the e-portfolio platform was assessed by descriptive tests (frequency and percentage). Data were analyzed by SPSS (ver. 23).

### Stage 3: exploration of educators’ experiences

In the qualitative stage, the experiences of the educators were collected using individual and semi-structured interviews and analyzed by a conventional content analysis introduced by Graneheim and Lundman [36].

### Participants

The participants were educators who participated in teaching e-portfolio. Maximum variation sampling was used to select the participants. Educators, who had the maximum and minimum participation in recording documents in the teaching e-portfolio, were purposefully selected and participated in this phase.

In the qualitative stage, 17 interviews were held. Nine women (52.94%) and 8 men (47.05%) were included in the study. The average work experience of the participants was 8 ± 2 years.

Qualitative data collection: The experiences of the educators were explored using individual and semi-structured interviews. The interview was coordinated with the participants in advance. The interviews were conducted by a trained interviewer (Ph.D. in medical education and qualified in qualitative research) in a quiet and secluded place at the EDC of their University.

#### Qualitative data collection

Before starting the interview, clarification was made regarding the benefits of conducting this research. The purpose of the research, the interview method, and the right of the individuals to participate in the study or refuse it was explained to the participants. They were assured about the recording of the interviews and the confidentiality of the information, and then informed consent was obtained from them. During data collection, all interviews were recorded.

To increase the credibility of the interview, an interview guide was developed. In this step, five experts in qualitative research who had experienced engagement with teaching portfolios assessed the validity of the interview questions in terms of necessity and relevancy to the purposes of the research. The validity of interview questions was confirmed from the perspective of the experts. According to the interview guide, interviews began with this main question: “Would you please tell me about your experience of engagement teaching e-portfolio?” what factors helped you to participate in this teaching e-portfolio? And what challenges did you experience in engagement with e-portfolio?. Some probing questions were asked for additional clarification to the answers given by the participants. Field notes were made during the interview by the interviewer. The process of selecting participants continued until a rich interpretation was reached and no new code emerged during the interviews (Saturation). Each interview lasted between 30 and 40 min.

#### Qualitative data analysis

A conventional content analysis introduced by Graneheim and Lundman was used for qualitative data analysis. Based on the conventional content analysis approach, the analysis process includes open code, categories, and themes [36].

Interviews were transcribed word by word and read line by line many times. Then, to extract the open codes, meaningful words, and short sentences were specified and coding emerged by taking notes in the margin of the text. Semantic units were extracted from the participants’ statements that expressed their experiences. Then the codes were merged and placed in categories based on semantic affinity. After organizing based on the relationship between them, the theme was formed.

#### Trustworthiness

In this study, the criteria of credibility, confirmability, transformability, and dependability were used [37]. The credibility of the data was achieved using semi-structured interviews, and prolonged engagement of the researcher with the data. The researchers engaged in the step for eight months. The extracted data and their analysis process were reviewed by the research team (peer check). The peer reviewers appraised the interviews, memo, and extracted findings, debated the researchers’ assumption, and asked questions about methods and interpretations. In addition, the text and related analyses were returned to the participants to ensure that the extracted findings explained were aligned with what they had experienced (member check). In the present study, to increase the confirmatory of the data, the interviews were conducted in a specific and continuous period with a full focus on the topic. The process of data analysis was carefully examined by experts with experience in the field of qualitative research. The auditor examines the data collection process, memos, and extracted codes to ensure the rigor of the data. In the audit process, the main concerns such as findings grounded in the data were addressed, the findings were logical, and the category structure was appropriate. In the current research, all the stages of the research were recorded in detail. To facilitate the transformability of the findings, a clear description of the context, method of selection, characteristics of the participants, data collection process, and data analysis process was provided. Rich description enables readers to make decisions about the applicability of the results to similar contexts [38].

#### Ethical considerations

In this research, the principles of information confidentiality and obtaining informed consent for interviews, recording conversations, and having the right to withdraw from the research at any time were taken into account.

## Results

### Stage 1: development of teaching e-portfolio

The teaching e-portfolio was formulated in 12 areas and 42 activities (Table 2).

**Table 2:** The components of the teaching e-portfolio

Area	Activities
Philosophy of education	Philosophy of education statement
Curriculum planning	Course/lesson plan development
	Designing and implementing (teaching in) continuous education programs
	Designing and implementing (teaching in) educators’ empowerment programs
	Designing and implementing continuous education program
Evaluation	Designing and implementing components of ‘a student assessment system’
	Designing and implementing high-stakes learners’ assessment methods (reasoning examination, workplace-based examinations, etc.)
	Participation in designing of regional/national/clinical competency-based examination
	Program and institution evaluation based on standards of international accreditation
	Participating in the internal evaluation of the educational institutes
	Participation in the external evaluation program
	Participation in the analysis of student exams
Teaching and learning	Using student-centered strategies and active learning methods in the classroom or educational fields
e-learning	Developing and conducting e-learning and blended learning (electronic, etc.)
Professional development in education	Participation in faculty empowerment workshops/training courses
Scholarly activities	Educational research
	Educational scholarship
Mentoring and counseling	Participation in mentoring programs
	Executive administration of extra-curriculum of students’ professional development
	Teaching in extra-curriculum of students’ professional development
Educational leadership	Participation in the design and implementation of processes, regulations, and instructions
	Participation in the preparation of long-term, prioritized and critical programs
Research in Education	Guiding and consulting theses in the field of research in education
	Presenting articles in education research field
	Reviewing medical education articles
	Reviewing projects of research in education
	Reviewing contents of virtual education
	Guidance and counseling on projects of research in education
Education reform project	Participating in the national project of education reform
Production of Scholarship of teaching and learning (Require to adherence of six criteria of Glassick (15))	Participation in curriculum development or reform of a curriculum
	Participation in curriculum planning or revision of an educational course
	Designing and implementing tools, methods, and processes for student assessment
	Designing and implementing tools, methods, and processes for faculty evaluation
	Internal and external evaluation of educational departments and programs
	Development of accreditation standards and indices
	Peer review of student assessment
	Designing and implementing an interactive/ student-centered teaching method
	Participation in the design and implementation of a change management project
	Designing and producing educational resource/educational tools
	Designing and producing study guides
	Participation in the design and implementation of the electronic educational systems
	Preparation of educational materials and learning assistance tools (film, educational application, and gamification, etc.)

### Stage 2: implementation of teaching e-portfolio and evaluation of the documentation quantitatively

In the investigated university, 200 educators were eligible to engage in an e-portfolio, and 148 educators registered in the e-portfolio (74%). Each educator could record one or more documents in different domains. The trend of educators’ engagement with teaching e-portfolio increased. A total of 1488 documents of educational activities were registered in the e-portfolio from 2018 to 2020, among which 55.24% of the activities achieved feedback in the peer review process, and 44.76% were scored in summative evaluation. The summary of the quantitative report of the e-portfolio is shown in Table 3.

**Table 3:** The quantitative report of engagement of educators in teaching e-portfolio

Area	Activities	The number of educators applying for e-portfolio	Number of documents applied in formative assessment	Number of documents applied in summative assessment	%
	Philosophy of education	69	69	69	100
Curriculum planning	Course/lesson plan Development	123	664	259	39
	Designing and implementing (teaching in) continuous education programs	6	14	14	100
	Designing and implementing (teaching in) educators’ empowerment programs	2	3	3	100
	Designing and implementing (teaching in) continuous education program	2	0	0	0
Evaluation	Participation in the design and implementation of components of ‘a student assessment system’	6	10	10	100
	Designing and implementing high stake learners’ assessment methods (reasoning examination, workplace-based examinations, etc.)	8	10	6	60
	Participation in designing of regional/national/clinical competency-based examination	9	12	5	41.66
	Program and institution evaluation based on standards of international accreditation	7	7	6	85.71
	Participating in the internal evaluation of the educational institutes	13	54	0	0
	Participation in the external evaluation program	28	132	0	0
Teaching-learning	Using student-centered strategies and active learning methods in the classroom or educational fields	34	110	43	39.09
e-learning	E-learning and blended learning (electronic, etc.)	3	127	123	96.85
Professional development in education	Participation in faculty empowerment workshops/training courses	5	81	81	100
Scholarly activities	Educational research	6	7	7	100
	Educational scholarship	13	13	4	30.76
Mentoring and counseling	Participation in students’ mentoring programs	8	10	4	40
	Executive administration of extra-curriculum of students’ professional development	3	13	12	92.30
	Teaching in extra-curriculum of students’ professional development	10	30	30	100
Educational leadership	Participation in the design and implementation of processes, regulations, and instructions	6	9	9	100
	Participation in the preparation of long-term, prioritized and critical programs	3	3	3	100
Education reform project	Participating in the national project of Education reform	10	10	10	100
Production of Scholarship of teaching and learning (Require to adherence of six criteria of Glassick (15))	Participation in curriculum development or reform of a curriculum	10	12	1	8.3
	Participation in curriculum planning or revision of an educational course	1	2	2	100
	Designing and implementing tools, methods, and processes for student evaluation	2	2	2	100
	Designing and implementing tools, methods, and processes for educators’ evaluation	2	3	3	100
	Internal and external evaluation of educational departments and programs	3	3	2	66.66
	Development of accreditation standards and indices	2	2	2	100
	Peer review of student assessment examinations	2	5	5	100
	Designing and implementing an interactive/ student-centered teaching method	5	5	1	20
	Participation in the design and implementation of a change management project	1	1	0	0
	Designing and producing educational products/educational tools	3	4	2	100
	Designing and producing study guides	11	11	11	100
	Total	416	1488	729	48.99

The results showed that most documents were in ‘Course/lesson plan development’ and ‘e-learning activities’. Minimum documents in the activity of research in education were reported.

### Stage 3: exploration of educators’ experiences

The experiences of the participants were explained in a theme including “fear and hope in utilizing of teaching e-portfolio”. This theme included three categories: “motivational roadmap for personal and professional development in the future”, “concern about the consequences of continuous monitoring” and “restriction of resources and capability as resistance sources”.

### Fear and hope in utilizing teaching e-portfolio

The positive and negative aspects of the participants’ experiences regarding engagement in the teaching e-portfolio were explained. A motivational roadmap for personal and professional development was explored as a positive experience for educators.

The challenges of engaging in the teaching e-portfolio were explained in two categories: “concern about the consequences of continuous monitoring” and “restriction of resources and capability as resistance sources”.

### A- motivational roadmap for personal and professional development

In this category, the experiences of the participants regarding the use of teaching e-portfolio as a planning tool for personal and professional development were explained. The participants believed that teaching e-portfolio by explaining the types of activities in different fields provides a platform for increasing awareness of excellent teaching and creating motivation to carry out these activities in the different domains.

#### A1- trigger of excellent teaching recognition

An assistant professor stated:

“When I saw the list of activities in the teaching portfolio, I became familiar with the activities that I can do to improve my teaching, and I got ideas from that list to plan my education.” (32-year-old female).

#### A2- self-regulation for purposeful planning

“I took help from the teaching portfolio to empower myself. The peer feedback helped me and I planned to participate in empowerment programs.“ (36-year-old female, assistant professor).

“The teaching portfolio helped me to have a purposeful plan to improve the quality of my education. I reviewed the assessment activities that I thought needed to be changed in my rotation. I planned to do them the next semester. Now, this activity was recorded in my profile.” (44-year-old male, professor).

### B- concern about the consequences of continuous monitoring

In this category, educators’ concerns about continuous monitoring by educational managers were classified. The participants stated that worrying about not having development activities in teaching e-portfolio and fearing its consequences in the process of promotion was their resistance factor to the expansion of teaching e-portfolio at the university.

#### B1- perceived stress from the consequences of the monitoring

An associate professor stated:

“I get very stressed when I see so many activities defined and I don’t take any action. I suspect that this lack of activity in this area and lack of documentation will cause problems for my promotion. So, I don’t prefer this system.” (48-year-old male).

#### B2- reluctance to monitor continuously

An associate professor said:

“I don’t like to be under monitoring all the time, especially regarding the quality of education, in which I don’t have much activity.” (48-year-old male).

An assistant professor stated:

“When I see comparative reports in my profile, I get stressed that my activities are so much less than others.” (40-year-old female).

### C - restriction of resources and capability as resistance sources

In this category, the factors affecting educators’ resistance to participating in teaching e-portfolio included the lack of skill in using technologies such as working with electronic systems, the time-consuming nature of participation in collecting documents and uploading them in the e-portfolio, as well as preparing structured forms as qualified documents. It was explained as acceptable in the peer review process.

#### C1- time-consuming involvement in teaching e-portfolio

An assistant professor said:

“It takes me a lot of time to complete the development activity report format. I prefer to do something more useful at this time”. (36-year-old female)

“The problem is probably due to this fact that we are not used to documenting our activities, but this system asks us to document, and this is difficult and time-consuming for me.” (44-year-old male, professor).

#### C2- weak technological literacy

“I prefer to have the same paper-based process for my work. Working with this electronic system causes a lot of trouble for me”. (56-year-old male, assistant professor)

“It is difficult for me to work with the e-portfolio, I do not know how to work with these electronic systems. I don’t even have time to learn” (52-year-old female, professor).

**Table 4:** The experiences of educators related to teaching e-portfolio

Open Code	Subcategory	Category	Theme
• Motivating to do more developmental activities • Better understanding of developmental activities in education • Encouraging to plan to participate in empowerment events to achieve developmental activities • Self-assessment opportunity • Planning for the future performance development • Encouraging to carry out developmental activities	Trigger of excellent teaching recognition	Motivational roadmap for personal and professional development in the future	Fear and hope in utilizing teaching e-portfolio
	Self-regulation for purposeful planning		
• Worrying about not having activities for all multiple areas • The stress of lack of registered documents and its impact on promotion • Resistance to the adoption of portfolio as a continuous monitoring tool based on the quality of education • Worrying about the consequences of the lack of appropriate activity in the field of development of the quality of education in grade and degree promotion	Perceived stress from the consequences of the monitoring	Concern about the consequences of continuous monitoring	
	Reluctance to monitor continuously		
• It takes time to complete the portfolio • Difficulty working with the electronic system • Preferring a manual and paper approach in data collection • The need to carry out administrative bureaucracy to collect acceptable documents • It is time-consuming to prepare reports and documents to present in the portfolio	Time-consuming involvement in teaching e-portfolio	Restriction of resources and capability as resistance sources	
	Weak technological literacy		

## Discussion

In this study, the teaching e-portfolio was developed from the perspective of SoTL concepts. The goal of the teaching e-portfolio was to assist educators to improve their teaching skills and excellent teaching through self-assessment, reflection, and continued learning, as well as, collect materials applied for faculty evaluation. The participants’ experience was explored in a theme, “fear and hope in utilizing teaching e-portfolio”, which was categorized into three classes: “motivational roadmap for personal and professional development in the future”, “concern about the consequences of continuous monitoring”, and “restriction of resources and capability as resistance sources”.

A teaching portfolio was introduced as a mechanism for evaluating the success rate of educators in activities involving teaching quality [6]. The results showed that the most registered documents were in the curriculum planning activities. The educators’ performance was influenced by the policies of the university. In our university, the process of instructional design and development of course plans in the faculties was determined as a requirement of teaching and assessed two years ago. In addition, a faculty development program and peer reviewing process related to the instructional design process have been implemented. This policy guided the performances of educators in the area. In the second place, activities in the e-learning area were considered, which can be due to the effect of the Covid-19 epidemic and the expansion of e-learning and distance learning methods in the universities. Therefore, micro and macro policymaking of educational administration and situational requirements directed educators toward the activities in the field of teaching quality.

Research in education and educational scholarship has been explained as a strategy for innovative methods in the education system. The results showed that the educators’ engagement rate was not acceptable in the area. The low engagement rate of educators in this area may be due to the specialization of the mentioned activities. These results indicated that the faculty development program must be considered in educators’ professional development programs. In addition, these activities need to provide approved documents in the councils confirmed by the vice-chancellor of education which could affect the rate of recorded documentation in these areas.

Most of the problems related to documentation accuracy were reported in the field of program evaluation. In this area, the certificate approved by the vice-chancellor of education was requested. So, the bureaucracies of certificate preparation may affect this issue which affected the low rate of engagement of educators and the high rejection rate due to inaccurate documentation. Similar to the present study, issues such as difficulty in storing and scoring documents and presenting inaccurate documents have also been mentioned in the study of Tisani and colleagues [39]. Since, the documentation in the e-portfolio was used for summative evaluation, in some areas; there is a need to register an approved certificate from an official authority, such as the vice-chancellor of education. The results indicated that the number of documents registered by the educators was higher in the areas where documentation requires less bureaucracy. It is suggested that in the design of the teaching e-portfolio aimed at summative evaluation, a balance between the validity of the documents and their accessibility should be considered.

The experience of the participants in using the portfolio was explained in the theme “fear and hope in utilizing teaching e-portfolio”. In line with our results, Hamilton used a teaching portfolio as bridging the gap from teacher to teacher educator. He explored the experiences of the educators in three categories: issues of identity and beliefs, scholarship in teacher education, and learning to teach how to teach [18]. So, the implementation of the teaching portfolio faced the incorporation of pros such as the professional development of educators, and cons for instant resistance and issues in the belief of educators and managers in medical education sciences systems.

In the present study, a motivational road map was explained to the positive experience of the educators in engagement with the teaching e-portfolio. The current results showed that e-portfolio as a tool for personal and professional development played an important role in creating motivation and persuasion for planning to carry out educational development activities. The participants became excellent teachers and believed that utilizing teaching e-portfolio has helped them get to know different areas of developmental activities and plan to do them in their education. For personal and professional development, a support mechanism is needed so that educators can evaluate their status and plan for their development in an individualized manner according to their perceived needs. The trigger for educators to enter this cycle is to understand the perspective of teaching quality and activities related to excellent teaching. This can be an important motivating factor to guide them in the cycle of self-evaluation, self-regulation, and planning to improve performance. The results of the present study showed that the creation of the teaching portfolio platform from the perspective of SoTL made the educators understand the gap between excellent teaching activities and plan to improve it. This experience of the participants is discussed in the trigger of the excellent teaching recognition category. Educators perceived the portfolio as a platform for purposeful planning to develop professional practice to achieve excellent teaching goals. Creating individualized learning opportunities through understanding needs, reflection about one’s performance, planning to meet perceived needs, receiving peer feedback, and understanding the results has created a motivational cycle for educators to be excellent teachers. They believed to be able to plan purposefully to participate in appropriate empowerment programs for the growth of teaching quality activities, such as mentoring and educational scholar programs [40, 41]. They explained teaching e-portfolio as a planning way to improve the quality of their education. Also, the participants considered e-portfolio as a tool for self-evaluation and reflection. They believed registration, peer review, and feedback played an important role in motivating them to participate in the activities of teaching quality. Likewise, Pelger and colleagues stated that writing a teaching portfolio had a positive effect on the development of educators. Integrating portfolio writing and peer feedback into educational programs encouraged educators to participate in developmental activities in education and created a new capacity for collaborative and reflective interactions. In addition, monitoring the quality of educational performance and writing educational portfolios facilitates educators’ achievements in the field of educational quality [21]. In line with our results, Hoekstra and Crocker showed using e-portfolio increased educators’ awareness of the areas of developmental activities and clear planning for their professional development [24]. In teaching activities, peer interactions and reflection are known as powerful mechanisms for the development of professional identity as an educator. Trautwein stated that the teaching portfolio through peer evaluation led to the professional development of educators and the improvement of their teaching practice. This process leads to the development of reflection strategies and peer evaluation to improve the quality of education [42].

From a managerial perspective, the traditional strategies that were mainly implemented in this institute [17] required reform. Then, a teaching portfolio was used to create a change in education and expand the use of innovative strategies in faculties. The educators who engaged in the teaching portfolio experienced the opportunity to familiarize themselves with excellent teaching activities to improve the quality of education in a comprehensive and classified manner. Moreover, the results showed the teaching e-portfolio raised awareness and motivating educators to achieve excellent teaching. In this study, the participation of the educators in the teaching portfolio was not mandatory to reduce the challenges of superficial and ineffective participation. This issue can also affect considering a portfolio as a support and motivational opportunity. In line with the present study, Deshpande introduced a portfolio as a tool for reformulating educational practice for the future and a source of motivation and self-regulation. Also, the participants of the study believed that using the teaching portfolio had created a new perspective on their work [13], which is similar to the present results. Likewise, Reece acknowledged the e-portfolio as an evaluation method provides a suitable opportunity for educators to reflect on their educational performance, and it also helps them to become familiar with the field of educational development and to be able to plan to improve their performance [6].

Concern about the consequences of continuous monitoring as a perceived issue was explained. In this category, the participants addressed their concerns about the function of the portfolio in monitoring their performance in educational quality activities. The participants believed that they were worried about being constantly monitored by system administrators. Although in various studies, monitoring is known as an advantage of the portfolio [7, 13, 21], in the present study it was explained as a factor of stress and worry. This finding can be caused by context-based challenges, such as the weakness of the evaluation systems in the university. In the studied university, the evaluation has been done twice a year and from the students’ point of view. In this process, many aspects of excellent teaching could not be evaluated. In addition, the concept of excellent teaching and the activities that a teacher did to achieve this goal were not monitored. Changing the evaluation culture among educators is associated with concerns and resistance. Resistance due to challenges of educator’s beliefs has been mentioned in various studies on the use of teaching portfolios in educational systems [17–19]. Planning to support and motivate educators can turn this perceived threat into an opportunity and be an important factor in guiding educators to participate in teaching quality improvement activities. The Little-Wienert and Shinkai in different studies debated the advantages of e-portfolio included: continuous monitoring of educational performance from the point of view of educational managers, peers, and the individual himself (herself), and the use of results for planning and policymaking at the system level. Although evaluating educational documents and creating a teaching portfolio is time-consuming and difficult, it supports educators to criticize their teaching and practical experiences in clinical or other settings, identify opportunities for improvement, and be encouraged to use performance for evaluation. Also, to make decisions regarding the promotion of educators, the evaluation results through the teaching portfolio provide a complete educational description of educators’ activities than the learner evaluation [5, 12].

Restriction of resources and capability as resistance sources was explained as an executive challenge in engaging in the teaching portfolio. Weak skills in using electronic systems and devices, the multiplicity of electronic systems for various activities of academic staff, and the time-consuming use of portfolios were the main reasons for educators’ resistance to using portfolios. Weakness in the use of electronic tools and technological literacy in the studied university was one of the reasons for educators’ resistance. Similar to the present study, Matthews and colleagues showed that the time consumed to complete the teaching portfolio was one of the concerns of participants in the teaching portfolio. They estimated the duration of 2 to 200 h to complete the teaching portfolio. In his study, educators’ unwillingness to spend time was explained as the key factor in the challenge of completing the portfolio [9]. In line with our results, Tisani explained some problems such as resistance against the portfolio, and problems related to portfolio evaluation and document evaluation [43].The implementation of traditional processes in universities is an important obstacle for educators to use new technologies. Farrell in his study, which examined the trend of using portfolios to e-portfolio in the world, in the 1980–2020 decade, stated that technologies emerged as mainstream in society and educational systems, and this made managers of their educational systems be adapted to technologies and use them in their systems. The development of electronic portfolios in line with the use of technology in educational systems in this decade is inevitable, and users of educational systems must develop the necessary ability to use technology [10].

The implications of these results in the investigated university were in the development of empowerment programs, teacher evaluation systems. In the field of empowerment, the results were used to plan for empowerment programs tailored to achieve excellent teaching activities and prepare educators to become educational scholars. Creating targeted empowerment opportunities such as SoTL training courses in the university, a training course with a mentoring and consulting approach was planned and its results were published in the author’s previous studies [40, 41, 44]. Also, individualized empowerment programs were planned for educators using virtual learning approaches, so that they can plan and implement personal and professional development processes based on their perceived needs. Results of this study were used to design a comprehensive system of faulty evaluation focusing on the use of education quality evaluation based on teaching portfolio, supporting mechanisms to remove the concern of continuous monitoring and planning for the application of the results of formative and summative evaluation in the institution level. The results will be published in future studies.

### Lessons learned

- The exchange of experiences related to the framework of excellent teaching, and the use of teaching portfolios in some developing countries where systematic mechanisms for evaluation are compiled, can be applied.
- In this study, the framework for evaluating the quality of teaching was developed from the perspective of SoTL. Since this framework has been developed by a scientific method, using the literature and viewpoints of different stakeholders, it can be used as a suggested framework for designing the teaching quality evaluation system by the teaching portfolio in other universities.
- The results of the study can be used as a step to design a comprehensive model for evaluating educational quality through teaching portfolios in universities of medical sciences.
- The Engagement in a teaching portfolio process can scaffold this process of developing SoTL among educators by facilitating the recognition of excellent teaching activities, self-reflection, and peer feedback, and encouraging quality improvement of their education process to achieve excellent teaching.
- Participants’ experiences regarding ‘hopes and concerns’ can be used in the design of teaching portfolios, such as:
- Educators like to improve their teaching competencies. The engagement with the teaching e-portfolio from the perspective of SoTL provided the opportunity to think about the subjects of quality improvement of their teaching, self-assessment, reflection, and self-regulation. As well, they encouraged to implement the methods and be involved in professional development as an excellent educator. The supportive mechanisms such as peer feedback, and learning opportunities based on their perceived needs suggest facilitating the development of educators.
- Educators prefer a simple, not time-consuming, and not stressful process for recording their achievements related to excellent teaching in e-portfolio. Developers of e-portfolio require to consider these factors in the development of components, and the process of involvement of educators with teaching e-portfolio.
- Educators perceived continuous monitoring as an unpleasant feeling. Also, understanding the follow-up process of personal development and the stages of becoming an excellent teacher can reduce the negative perception resulting from continuous monitoring among educators and highlight the motivating factors for their personal and professional development. Therefore, the application of the results of a formative and summative evaluation in tenure, promotion, and other tangible benefits for educators requires planning by educational managers.
- Electronic platforms and technology systems require being user-friendly, collaborative, and secure. The e-portfolio should minimize repetitive work and sync with other platforms for educators’ evaluation and promotion to reduce uploading the same documentation separately onto multiple platforms. An academic staff that is familiar with the process of e-portfolio may support and facilitate the engagement of educators with the teaching e-portfolio.
- Policies of the university about the education and methods of the evaluation of the educators and supportive mechanism is important in persuading them to use teaching e-portfolio for professional development to become excellent educator.
- Teaching portfolios provide informal and individualized opportunities for self-evaluation, self-regulation, and planning for educators’ personal development, which is expected to facilitate recognition and achievement of excellent teaching.
- Investigating the long-term impact of participating in teaching portfolios on the teaching performance of educators and the formation of their identity as excellent teachers is suggested in future studies.

### Limitations

Implementation of a teaching portfolio could not be mandatory for all educators due to a concern of superficial and ineffective use. The quantitative findings may be influenced by the voluntary participation of the educators. This study explored the experiences of educators by the qualitative method in one university, so the generalizability of the qualitative results is limited.

## Conclusion

In this study, the teaching e-portfolio was developed from the perspective of scholarship of teaching and learning concepts. The results showed participation rate of educators was at an acceptable level. The participation of educators in the area of educational planning and e-learning was reported at the highest level. The educational policies and educational situations were directed to the education quality activities of the educators. The educators perceived a teaching e-portfolio as a two-faceted tool that can provide a road map for their personal and professional development and direct their activities of teaching quality. As well, the teaching e-portfolio was considered a tool for continuous monitoring and detection of the inefficiency of teaching quality activities from the viewpoints of educators. This perception, along with limited resources such as time, weak technological literacy, and difficulty in working with electronic devices and systems, led to resistance from educators to use teaching e-portfolio.

## Data Availability

The datasets used and/or analyzed during the current study are available from the corresponding author upon reasonable request.
